# Uniaxial Magnetization Performance of Textured Fe Nanowire Arrays Electrodeposited by a Pulsed Potential Deposition Technique

**DOI:** 10.1186/s11671-017-2367-3

**Published:** 2017-11-21

**Authors:** C. Neetzel, T. Ohgai, T. Yanai, M. Nakano, H. Fukunaga

**Affiliations:** 1 Graduate School of Engineering, Nagasaki University, Bunkyo-machi 1-14, Nagasaki, 852-8521 Japan; 2 JSPS, Kojimachi 5-3-1, Chiyoda-ku, Tokyo, 102-0083 Japan; 3 Formerly: Technische Universität Darmstadt, Fachbereich Material- und Geowissenschaften, Alarich-Weiss-Strasse 2, 64287 Darmstadt, Germany

## Abstract

Textured ferromagnetic Fe nanowire arrays were electrodeposited using a rectangular-pulsed potential deposition technique into anodized aluminum oxide nanochannels. During the electrodeposition of Fe nanowire arrays at a cathodic potential of − 1.2 V, the growth rate of the nanowires was ca. 200 nm s^−1^. The aspect ratio of Fe nanowires with a diameter of 30 ± 5 nm reached ca. 2000. The long axis of Fe nanowires corresponded with the <200> direction when a large overpotential during the on-time pulse was applied, whereas it orientated to the <110> direction under the potentiostatic condition with a small overpotential. By shifting the on-time cathode potential up to − 1.8 V, the texture coefficient for the (200) plane, TC_200_, reached up to 1.94. Perpendicular magnetization performance was observed in Fe nanowire arrays. With increasing TC_200_, the squareness of Fe nanowire arrays increased up to 0.95 with the coercivity maintained at 1.4 kOe at room temperature. This research result has opened a novel possibility of Fe nanowire arrays that can be applied for a new permanent magnetic material without rare-earth metals.

## Background

Nanowire arrays with a high surface area show novel physical properties and are considered for applications in numerous industrial fields such as the fabrication of electronic and magnetic devices. The preparation processes include template-free methods [1–3] and template-based methods [4–7]. The template-based method using nanochannel structures such as ion track-etched foils or aluminum oxide membranes [8] is a promising technique to achieve precise length and diameter scales. In this method, the one-dimensional shape is directly adapted to the pore dimension of the membrane using electrodeposition techniques. Because of the possibilities of achieving high porosities and pore aspect ratios at low costs, anodic aluminum oxide (AAO) exhibits many advantages compared with other membrane materials.

Some researchers have reported that Ni, Co, and Fe nanowires can be electrodeposited in the nanochannel of membranes on metals [9, 10]. Hu et al. reported that Fe nanowire arrays can be electrodeposited by applying the direct current electrodeposition technique using acidic chloride bath [11]. In their report, the effects of diameter and crystal orientation of Fe nanowires on the low temperature magnetic properties were examined. They revealed that the coercive force increased up to ca. 2 kOe at 5 K when decreasing the diameter of Fe nanowires down to ca. 30 ± 5 nm. They also found that the magnetic squareness of Fe nanowires with (200)-orientation was larger than that with (110)-orientation. Irfan et al. reported the effects of post-annealing on the magnetic properties of Fe nanowires with the aspect ratio of ca. 80–100, which are potentiostatically electrodeposited at − 1.1 V vs. SCE [12]. Cornejo et al. also reported that Fe nanowires can be prepared using AC electrodeposition at a cell voltage of 15 V. They revealed that the length of Fe nanowires is approximately 3–5 μm and the aspect ratio is ca. 100 [13]. The magnetic force of a permanent magnetic film increases with increase in the surface magnetic flux density. The magnitude of the surface magnetic flux density depends on the thickness of a magnetic film, while the magnetic coercive force of a permanent magnetic film increases with decrease in the diameter of magnetic crystal grains. Hence, for a permanent magnet application, a high aspect ratio of Fe nanowires is required in the industrial production line. However, in the previous works, the aspect ratio of Fe nanowires did not reach 1000. Recently, we have reported that Co nanowires with an aspect ratio of more than 2000 can be electrodeposited by a potentiostatic electrodeposition technique using AAO nanochannels with a large aspect ratio [8]. In our previous study, to obtain the Co nanowires with a large aspect ratio, the electrolytic solution temperature was kept at higher than 80 °C and the cathodic overpotential was kept smaller than 0.2 V to enhance the growth of Co nanowires and to avoid hydroxide formation in the small AAO nanochannels. However, in the case of Fe electrodeposition, a high temperature solution will accelerate the hydroxide formation in the AAO nanochannels and inhibit the growth of Fe nanowires. Potentiostatic electrodeposition in a small cathodic overpotential range at room temperature will cause a small growth of Fe, while the pulsed potential deposition technique, which enables the achievement of a large cathodic overpotential, will prompt a large growth of Fe nanowires with a large aspect ratio. Hence, in this study, we fabricated Fe nanowire arrays with the aspect ratio up to 2000 and examined the effect of the deposition overpotential, which can be controlled by potentiostatic and pulsed potential deposition techniques, on the crystal orientation and magnetic performance of the nanocomposite films with Fe nanowires.

## Experimental

**Fig. 1:**
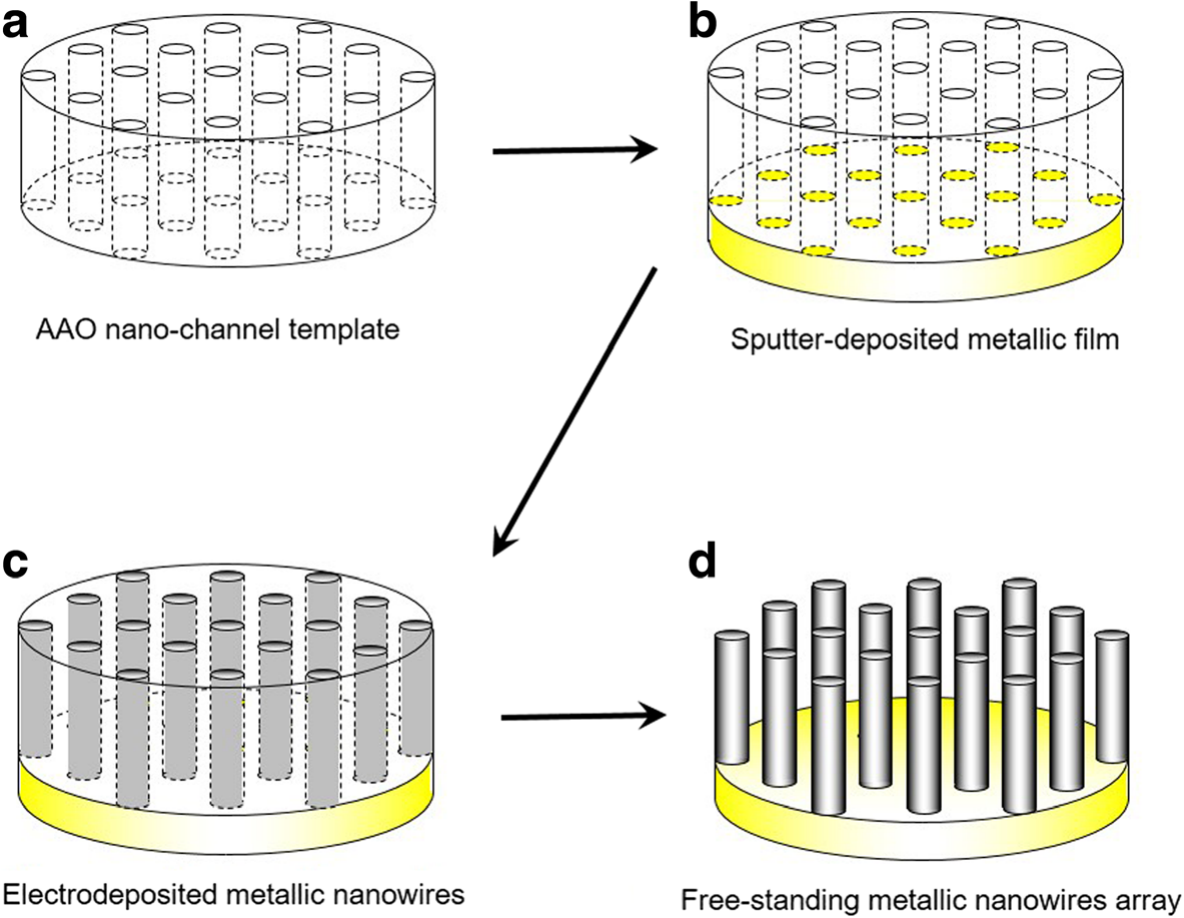
Fabrication process of free-standing metallic nanowire array. **a** Anodized aluminum oxide nanochannel template, **b** sputter-deposited metallic film, **c** electrodeposited metallic nanowires, and **d** free-standing metallic nanowire array

AAO membranes were manufactured by anodization of a pure aluminum rod (99.99%) with a diameter of 10 mm. First, a cross section of an aluminum rod was mechanically and subsequently electrochemically polished in an ethanol solution containing 20% perchloric acid while applying an anodic current density of 3.0 A cm^−2^ for 120 s. Next, the anodization was conducted in 0.3 mol L^−1^ oxalic acid at 12 °C for 22 h in a one-step process by applying a constant cell voltage of 30 V. During the anodization, the electrolytic solution was stirred by a magnetic stirrer at a stirring rate of 250 rpm. The membrane was obtained by immersing the rod in an ethanol/perchloric acid mixture and applying a voltage of 40 V for 3 s [14]. Finally, as shown in Fig. 1a, an AAO nanochannel template was prepared by separation from an aluminum rod. Prior to electrodeposition, a thin gold layer (ca. 200 nm in thickness) was sputter-deposited on one side of the membrane in an ion sputtering device, JFC-1600 (JEOL, Tokyo, Japan), by applying a current of 10 mA for 900 s. The electrode was then prepared by attaching the gold side of the AAO foil to a copper plate with silver paste. The iron deposition was conducted in a 0.05 mol L^−1^ iron sulfate heptahydrate solution (pH 2) at a temperature of 30 °C. A thin gold wire served as a counter electrode and Ag/AgCl as a reference electrode. When potentiostatic deposition was to be used, a cathode potential of − 1.2 V referred to Ag/AgCl was applied. In contrast, the rectangular-pulsed potential deposition was conducted at − 1.5 V (or − 1.8 V) during the on-time pulse (*t*_on_ = 0.1 s) and at − 1.0 V during the off-time pulse (*t*_off_ = 1.0 s).

After the electrodeposition, AAO membranes were dissolved by immersing the samples in 5 mol L^−1^ NaOH aqueous solution to obtain Fe nanowires. In the alkaline solution, alternation of morphology or crystal orientation of Fe nanowires was not observed. Structure and crystallographic orientation of Fe nanowire arrays were characterized by field emission scanning electron microscopy (JEOL-JSM-7500FA, accelerating voltage 5 kV) and transmission electron microscopy (JEOL-JEM-ARM200F, accelerating voltage 200 kV) as well as by X-ray diffraction (XRD: Rigaku-SmartLab, Cu K_α_ source). Magnetic properties of Fe nanowire arrays were investigated using a vibrating sampling magnetometer (VSM) at room temperature. The hysteresis loops were obtained in the magnetic field which was applied along the perpendicular and in-plane directions with the external magnetic fields up to 10 kOe. The perpendicular direction corresponds to the long axis of Fe nanowires, which is perpendicular to the plane of a membrane film, while the in-plane direction corresponds to the short axis of Fe nanowires, which is in-plane with a membrane film.

## Results and Discussions

### Electrodeposition of Fe Nanowire Arrays

**Fig. 2:**
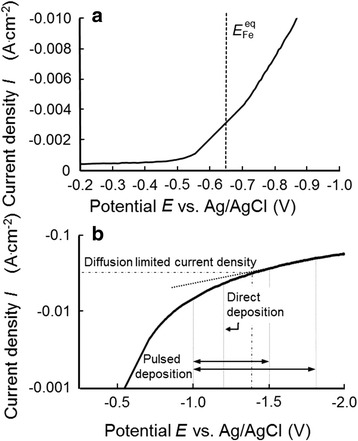
**a** Cathodic polarization curve from a 0.05 mol L^−1^ FeSO_4_ electrolyte at 30 °C and **b** Tafel plot of the polarization curve. Scan rate was fixed to 30 mV/s

Figure 2a shows a cathodic polarization curve linearly scanned from − 0.2 V to − 1.0 V at a rate of 30 mV s^−1^ and at a solution temperature of 30 °C. The current density was calculated using the area of the whole membrane (ca. 0.28 cm^2^), which was in contact with the electrolytic solution. Constant small current densities of approximately 4.5 × 10^−4^ A cm^−2^ were measured from − 0.2 to − 0.5 V, whereas a sharp increase was observed at − 0.55 V. Equilibrium potential of Fe/Fe^2+^ in the experimental condition can be estimated to be ca. − 0.68 V vs. Ag/AgCl according to Nernst’s equation (*E* ^eq^ = *E* ^0^ + *RT*/*nF* × ln*M* ^n+^/*Mn* ^0^, where *E* ^0^ = − 0.64 V vs. Ag/AgCl, *R* = 8.3 J K^−1^ mol^−1^, *T* = 303 K, *n* = 2, *F* = 96,485 C mol^−1^, and *M* ^n+^/*Mn* ^0^ = 0.05). Therefore, the observed slope rise in Fig. 2a is mainly attributed to hydrogen evolution, which usually takes place as a competitive reaction with metal depositions in aqueous solution [15, 16]. The pore may not be fully filled with hydrogen gas enabling penetration of Fe ions into the pore. Hence, the temporarily trapped hydrogen gas will be pushed to the outside of pores by growing of metal deposits. As shown in Fig. 2a, in the region of approximately − 0.70 V, the slope of the i-V curve increased slightly, which implies the start of Fe deposition. Figure 2b shows a Tafel plot, which was obtained by logarithmically plotting the current of Fig. 2a in the potential ranging from − 0.5 to − 2.0 V. As shown in Fig. 2b, the slope of the curve decreased with increasing cathodic overpotential. In the potential range lower than − 1.4 V, the slope reached a constant. This phenomenon was caused by the electrophoretic migration mechanism for metal cations in the pore. It is well known that the optimum deposition potential for growing nanowires can be determined by a cathodic polarization curve obtained in a wide cathode potential regime [17]. Usually, the optimum deposition potential should be selected to a potential region which is nobler than that controlled by electrophoretic migration. Considering the results obtained from Fig. 2, the optimum cathode potential for growing Fe nanowires inside the pores of the AAO membranes was determined to be − 1.2 V for potentiostatic deposition. In contrast, in the rectangular-pulsed potential deposition, cathode potential during the on-time pulse was adjusted to − 1.5 or − 1.8 V to achieve large overpotential for a short time, whereas the cathode potential during the off-time pulse was fixed to − 1.2 V to avoid the dissolution of deposited Fe.

**Fig. 3:**
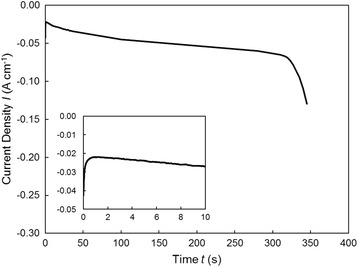
Time-dependence of current density during Fe nanowire growth at − 1.2 V. The beginning of the deposition process is also shown in the inset

Figure 3 depicts an example for the potentiostatic deposition of Fe nanowires at − 1.2 V. At the initial stage, the decrease in current density was due to the decrease in concentration of cations such as Fe^2+^ and H^+^ in the pores. Subsequently, the current density showed an almost steady value because of the stable supply of cations from the bulk of solution to the pores [18]. In general, the end of the nanowire growth is reflected by a rapid increase in the current density because of a cap growth on top of the membrane. This is simultaneously accompanied by a continuous increase in the electrode area [19]. In our experiments, we utilized a membrane thickness of ca. 60 ± 5 μm that is the same as the lengths of the Fe nanowires. With a filling time (time difference between the start and the sudden increase in the current density according to Fig. 3) of 300 s, the growth rate was estimated to be ca. 200 nm s^−1^.

**Fig. 4:**
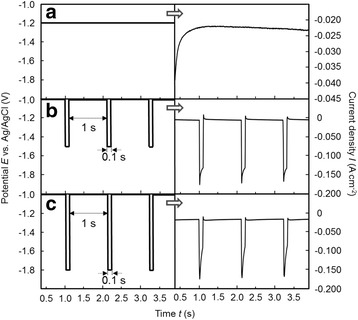
Time dependence of applied potentials (left-hand side) with observed current density (right-hand side) during Fe nanowire deposition. **a** Potentiostatic deposition at − 1.2 V, **b** pulsed potential deposition with the on-time potential of − 1.5 V, and **c** pulsed potential deposition with the on-time potential of − 1.8 V

Figure 4 shows representative examples of applied potential patterns (left-hand side) and the observed current density response (right-hand side) over a period of 4 s. In the case of the potentiostatic deposition (Fig. 4a), an initial decrease of the current density was observed, and the current reached a constant value of less than 2.5 × 10^−2^ A cm^−2^ in the linear electrophoretic migration-controlled growing regime during the homogenous filling of the pore channels. In contrast, in the rectangular-pulsed potential deposition case, a slight difference was observed on the current density responses for the on-time pulse when the potential was fixed to − 1.5 V (Fig. 4b) or − 1.8 V (Fig. 4c) for 0.1 s. According to Fig. 4b, c, current density responses during the on-time pulse revealed almost the same value. However, during the off-time pulse, a clearly different pattern was observed. Figure 4b shows that the anodic current was observed during the off-time pulse and the cathodic current reached a constant value of approximately − 6.2 × 10^−3^ A cm^−2^. In contrast, according to Fig. 4c, a constant current density of − 1.8 × 10^−2^ A cm^−2^ was observed during the off-time pulse. For both samples, nanowire growth was mainly promoted during the on-time pulse, which led to a different crystallization behavior compared with the potentiostatic deposition. In particular, the pulse time and the amplitude are crucial features for crystallization behaviors. Therefore, these pulse parameters will strongly influence the physical properties of the electrodeposited Fe nanowires. In general, crystallization processes occur in competition of two routes in which either assembling of old crystals or the formation of new ones takes place. These processes are mainly influenced by surface diffusion rates, i.e., the movement of ad-atoms to growing steps [20]. In this study, Fe nanowire arrays are prepared at high current densities during the on-time pulse using the pulsed potential deposition technique. In contrast, at − 1.0 V during the off-time pulse, Fe^2+^ ion concentration at the surface will be recovered by a decrease in the reduction rate of Fe^2+^ ions. When the potential shifts to − 1.5/− 1.8 V at *t*_on_, recovered Fe^2+^ concentration provides enough large cathodic (deposition) current as seen in Fig. 4 [17].

### Structure and Crystallographic Orientation of Fe Nanowire Arrays

**Fig. 5:**
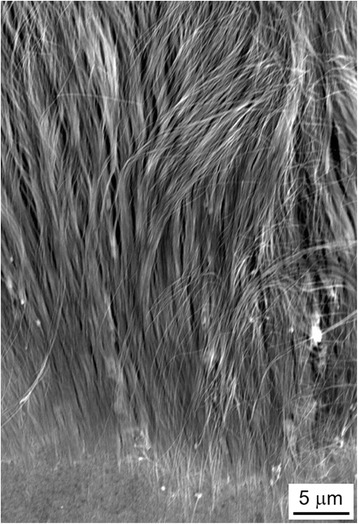
SEM cross-section image of arrayed Fe nanowires separated from a AAO membrane

**Fig. 6:**
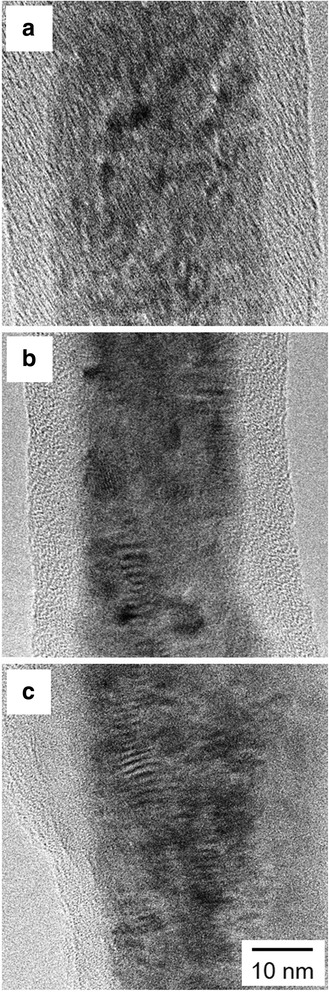
TEM bright-field images of Fe nanowires separated from a AAO membrane. **a** Potentiostatic deposition at − 1.2 V, **b** pulsed potential deposition with the on-time potential of − 1.5 V, and **c** pulsed potential deposition with the on-time potential of − 1.8 V

Figure 5 shows a SEM cross-section image of arrayed Fe nanowires separated from a AAO membrane. The one-dimensional structures were densely packed, and each nanowire lay in a parallel direction. Figure 6 shows the TEM bright-field images of Fe nanowires separated from a AAO membrane. These samples were prepared by potentiostatic deposition at − 1.2 V (Fig. 6a), pulsed potential deposition with the on-time potential of − 1.5 V (Fig. 6b), and pulsed potential deposition with the on-time potential of − 1.8 V (Fig. 6c). The diameter of Fe nanowires was also estimated to be ca. 30 ± 5 nm by a TEM image in Fig. 6. Under the previously described anodization condition (30 V, 12 °C, and 22 h), the membrane also exhibited an average pore diameter of ca. 30 ± 5 nm [8]. Considering a membrane thickness of ca. 60 ± 5 μm, an ultra-high aspect ratio of 2000 was achieved in our experiment. TEM images of the samples, which were prepared by pulsed potential deposition (Fig. 6b, c), revealed that some crystal defects existed in the structure. These crystal defects can be caused by the internal tensile stress, which originate from the large overpotential for Fe deposition during the on-time pulse.

**Fig. 7:**
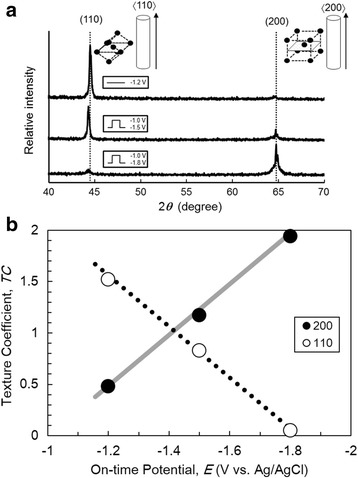
Crystal orientation and morphology of Fe nanowire arrays. **a** X-ray diffraction patterns. **b** On-time potential dependency calculated texture coefficients from x-ray diffraction patterns

Figure 7a shows X-ray diffraction patterns of bcc Fe nanowire arrays. The results confirm that the crystallographic orientation is highly sensitive to the variation of deposition parameters. Among bcc crystal planes, (110) is mostly close-packed by atoms and the surface energy is minimum. Hence, in the electrodeposition with small overpotential, (110) orientation will occur preferentially [21]. Potentiostatic deposition led to a clearly enhanced emergence of the (110) peak. In comparison, the pulsed deposition technique, which can realize a cathode potential less than − 1.8 V, resulted in a preferred (200) orientation. The (200) peak increased with increasing deposition potential during the on-time pulse. The (110) peak almost vanished for Fe nanowire arrays prepared with the on-time pulse potential of − 1.8 V. Figure 7a also revealed a shift of the (110) peak and a shoulder of the (200) peak for the Fe nanowires deposited by pulsed deposition with respect to those grown by potentiostatic deposition. The peak shift and the shoulder may have been caused by the internal tensile stress which results in emerging of crystal defects in the structure as shown in Fig. 6b, c. Hence, the peak shift and the shoulder originated from the large overpotential for Fe deposition during the on-time pulse.

The texture coefficient (TC) is calculated using the Harris formula [22].

1 $$ \mathrm{TC}\left(h,k,l\right)=\frac{I\left({h}_i{k}_i{l}_i\right)/{I}_0\left({h}_i{k}_i{l}_i\right)}{1/N\times {\sum}_{j=1}^N\left(I\left({h}_j{k}_j{l}_j\right)/{I}_0\left({h}_j{k}_j{l}_j\right)\right)} $$

Equation (1) describes the analysis of the relative peak intensities dependent on *I(h*_*i*_ *k*_*i*_ *l*_*i*_ *)*, i.e., the intensities observed from *h*_*i*_ *k*_*i*_ *l*_*i*_ lattice planes of the sample, and *I*_0_ *(h*_*i*_ *k*_*i*_ *l*_*i*_ *)* denotes the intensities of a standard Fe powder. *N* is the number of diffraction planes considered for the determination of TC. Figure 7b shows the relationship between the TCs calculated for (200) and (110) planes and the electrodeposition potential of Fe nanowire. Potentiostatic deposition led to a preferred (110) orientation with TC_110_ of 1.52. In this case, the long axis of the nanowire was <110>. In contrast, pulsed deposition with the on-time pulse potential of − 1.5 V resulted in TCs of almost 1 for both (110) and (200) planes denoting randomly oriented crystals in the deposit. Furthermore, Fe nanowires prepared with the on-time pulse potential of − 1.8 V clearly showed (200) orientation with TC_200_ of 1.9.

### Perpendicular Magnetization of Fe Nanowire Arrays

**Fig. 8:**
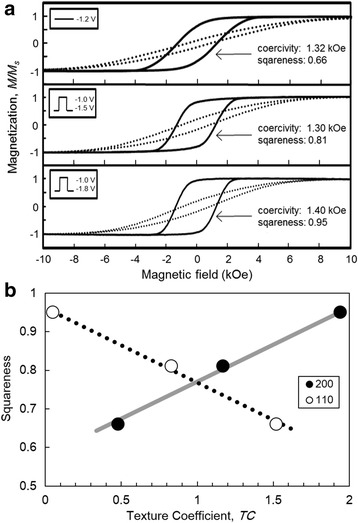
Magnetic properties of Fe nanowire arrays. **a** Magnetic hysteresis loops with the magnetic field in perpendicular (solid line) and in-plane (dotted line) direction. **b** Relationship between squareness and TC_200_ and TC_110_

Figure 8 shows magnetization curves of Fe nanowire arrays. Any corrections of paramagnetic or diamagnetic contributions were not performed for the shown hysteresis loops. According to Fig. 8a, all structures showed a pronounced magnetic anisotropy, which was reflected by different potential waveforms for different measurement directions (perpendicular direction: solid line and in-plane direction: dotted line). The samples prepared by potentiostatic deposition as well as pulsed deposition with the on-time pulse potential of − 1.5 V had almost the same perpendicular coercivity of 1.3 kOe. A slightly increased coercivity of 1.4 kOe was measured for Fe nanowire arrays fabricated by the on-time pulse potential of − 1.8 V. However, in particular, the squareness (defined as the ratio of the remnant to saturated magnetization) gradually increased with increasing TC_200_. The hysteresis curve clearly changed from an oblate to a square waveform. Similarly, as shown in Fig. 8b, an increase in squareness from 0.65 to 0.95 was achieved.

It is well known that the crystalline orientation can be modified by deposition conditions such as the choice of potentiostatic and pulsed potential deposition [23]. In particular, pulsed deposition is a powerful technique to improve uniform growth avoiding the formation of large and randomly oriented crystallites [23]. Furthermore, the low pH value of the electrolyte should be considered. As discussed above, fabrication of Fe nanowires is preceded by the simultaneous reduction of hydronium ions, which results in local pH changes inside the pores of the AAO membrane [24]. Moreover, hydrogen can be easily absorbed in the deposit, significantly influencing its crystallinity [25]. In this case, the metal Fe deposition rate might be considerably reduced. It is well known that the hard axis for the magnetization of bcc Fe is in the <110> direction, which results in reduction of the squareness in magnetization. This uniaxial magnetization behavior of Fe nanowire arrays was confirmed in this study. Yang et al. reported that Fe nanowires, which were fabricated using potentiostatic electrodeposition at a constant cell voltage of 1.5 V, had a random orientation without texture [11]. Irfan et al. also reported that Fe nanowires, which were potentiostatically electrodeposited at − 1.1 V vs. SCE, exhibited a non-textured orientation and the coercive force of ca. 0.5 kOe [12]. Cornejo et al. also reported that Fe nanowires, which were prepared using AC electrodeposition at a cell voltage of 15 V, had a random orientation without texture and the squareness of ca. 0.5 [13]. In the current study, Fe nanowires with an aspect ratio of 2000, which were electrodeposited using a rectangular-pulsed potential deposition technique to control the crystal orientation, had a strong texture with (200) orientation. The textured Fe nanowires exhibited the coercive force of ca. 1.4 kOe and the squareness of ca. 0.95. Hence, we demonstrated that the rectangular-pulsed potential deposition technique can control the crystal orientation and aspect ratio of Fe nanowires, leading to excellent magnetic properties.

## Conclusion

The degree of overpotential during the potentiostatic and the pulsed potential deposition significantly affected the crystal orientation and the magnetization performance of high aspect ratio Fe nanowire arrays. According to the determination of the texture coefficients, potentiostatic deposition at a cathode potential of − 1.2 V led to a preferred (110) orientation, whereas pulsed techniques resulted in either randomly oriented crystallites or a (200) orientation by applying on-potentials of − 1.5 and − 1.8 V, respectively. Magnetic hysteresis loops in perpendicular and in-plane directions to the membrane surface showed a strong magnetic anisotropy because of the high aspect ratios (approximately 2000) of all considered Fe nanowire arrays. Therefore, the crystalline orientation and the shape anisotropy are the most important factors controlling the magnetic properties. The coercivity obtained in the magnetic field for the long axis direction of Fe nanowire arrays with a preferred (110) orientation was 1.3 kOe. This value slightly increased to 1.4 kOe for the nanowires with strong (200) orientation. In contrast, the squareness obtained from Fe nanowire arrays with a preferred (200) orientation significantly increased up to 0.95 from 0.65 with increase in TC_200_. This study illustrates the feasibility of improving the magnetic properties of Fe nanowire arrays by controlling the degree of overpotential during electrodeposition.
